# Outcome of follow-up computed tomography of suspected occult scaphoid fracture after normal radiography

**DOI:** 10.1007/s10140-024-02307-0

**Published:** 2025-01-08

**Authors:** Mats Geijer, Eirikur Gunnlaugsson, Linnea Arvidsson, Elin Österhed, Magnus Tägil

**Affiliations:** 1 Department of Radiology, Institute of Clinical Sciences, Sahlgrenska Academy, University of Gothenburg, Gothenburg, Sweden; 2 Institute of Clinical Sciences, Lund University, Lund, Sweden; 3 Department of Medical Imaging and Physiology, Skåne University Hospital, Lund, Sweden; 4 Department of Orthopaedics, Institute of Clinical Sciences, Lund University, Lund, Sweden; 5 Skåne University Hospital, Lund, Sweden

**Keywords:** Scaphoid fracture, Computed tomography, Occult fracture, Diagnosis

## Abstract

**Purpose:**

To evaluate the rate of missed scaphoid fractures on follow-up computed tomography (CT) for suspected occult scaphoid fracture after normal radiography with residual radial-sided wrist pain.

**Methods:**

In a retrospective analysis, wrist CT during a five-year period was analyzed. The CT examinations and radiological reports were re-evaluated. Available clinical findings and radiologic follow-up performed during a period of a minimum of three years served as outcome reference.

**Results:**

In total, 178 examinations had been performed on 174 patients for suspect scaphoid fracture, 67 men and 107 women, showing 15 and 6 scaphoid fractures, respectively; a statistically significant sex difference (*p* = 0.0024). In 157 examinations, no scaphoid fracture was detected on CT, instead 29 other wrist or carpal bone fractures were found. On follow-up, no missed scaphoid fractures were found. Before CT, 124 of the 157 patients had been treated with a cast. After CT, 35 patients continued with cast treatment for a median of 14 days.

**Conclusions:**

CT appears to be a reliable method for evaluating suspect scaphoid fracture as part of a diagnosis-treatment regimen including pain immobilization with a plaster cast.

## Introduction

The scaphoid is the most commonly injured carpal bone [1–5]. Most fractures heal well after conservative or surgical treatment, but complications such as non-union or avascular necrosis (AVN) may lead to scapho-lunate advanced collapse (SLAC) or scaphoid non-union advanced collapse (SNAC). Primary imaging with radiography of the wrist with supplemental special projections of the scaphoid will detect most fractures, but a not insignificant number will remain occult, and may, if undetected, lead to complications [6]. Different prevalence figures of radiographically occult scaphoid fracture have been reported according to varying inclusion criteria and radiographic technique. On average, the figure is 16% in meta-analyses [7–9]. Supplementary imaging can be done with several modalities such as planar bone scintigraphy [10–12], single photon emission computed tomography (SPECT/CT) [13], magnetic resonance imaging (MRI) [14], CT [15, 16], cone-beam CT (CBCT) [17], tomosynthesis [18] and ultrasound [19]. MRI is today by many regarded as the reference standard [20] providing both anatomic and physiologic information. In a recently published review [21] MRI is recommended as the primary follow-up imaging modality in suspected occult scaphoid fracture. But by relying too much on bone bruise for MR diagnosis overtreatment of purely trabecular fractures is possible [20]. CT is often seen as less sensitive since only cortical fractures but not bone bruise can be detected, however the pooled sensitivity and specificity of CT in diagnosing a scaphoid fracture has been reported as 93% and 99%, respectively [22, 23]. In contrast, a recent comparative study reported 25% scaphoid abnormalities on MRI after a negative CT [24].

To follow up a patient with suspect occult scaphoid fracture, i.e. residual pain after a wrist trauma and normal radiography including special scaphoid views, various regimens may be undertaken [25]. Even though MRI is the most sensitive modality, and combines physiologic and anatomic data, there is no consensus in the literature on the most cost-effective way for follow-up.

One of the drawbacks of MRI is the reduced ability to display a non-displaced cortical fracture, which is one of the strengths of CT. CT, on the other hand, suffers from the inability to display clearly bone marrow edema, which is one of the strengths of MRI. Comparisons between these two modalities are few [16, 23, 26], usually involving few patients, without clear-cut results. One analysis of comparative studies concluded that both modalities have flaws, and both are better at ruling out fractures than confirming them [26]. One way of resolving this issue would be to evaluate CT performance at an institution before MRI was widely accepted as second-line imaging in suspect occult scaphoid fracture. Thus, it was hypothesized that in a situation without available MRI, wrist CT performed on a whole-body CT scanner would have the ability to detect all cortical scaphoid fractures with a potential for future displacement.

The purpose of the current study was to evaluate whether CT of suspect occult scaphoid fractures was robust enough to be trusted in clinical practice by retrospectively evaluating the final outcome after negative scaphoid CT, i.e. whether CT of suspect occult scaphoid fractures detects all scaphoid fractures with a potential for future displacement.

## Material and methods

### Ethical approval

Ethical approval was obtained from the Swedish Ethical Review Authority (2019–01463), and the need for informed consent was waived.

### Patients

In a retrospective analysis, all CT examinations of the wrist and hand performed during five years, from January 1, 2006, to December 31, 2010, at a university hospital were collected from the radiology information system (RIS). By manual perusal of the request forms, all wrist and scaphoid CT examinations performed for evaluation of a suspect occult scaphoid fracture were selected. Inclusion criteria: CT performed for residual post-traumatic radial-sided wrist pain and suspected scaphoid fracture with previous clinical radiography negative for scaphoid fracture and CT performed at least three years before study inclusion. Exclusion criteria: Patients referred to wrist CT for other reasons than suspected occult scaphoid fracture and patients treated for scaphoid fracture, including those with MRI-verified scaphoid fracture or bone bruise treated with a cast. During the inclusion period, CT was the routine advanced imaging modality of choice for occult scaphoid fracture at the study institution.

**Fig. 1:**
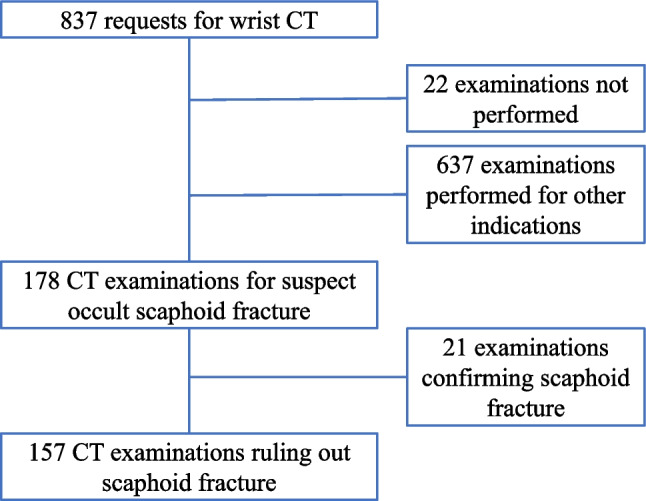
Inclusion flow chart

**Fig. 2:**
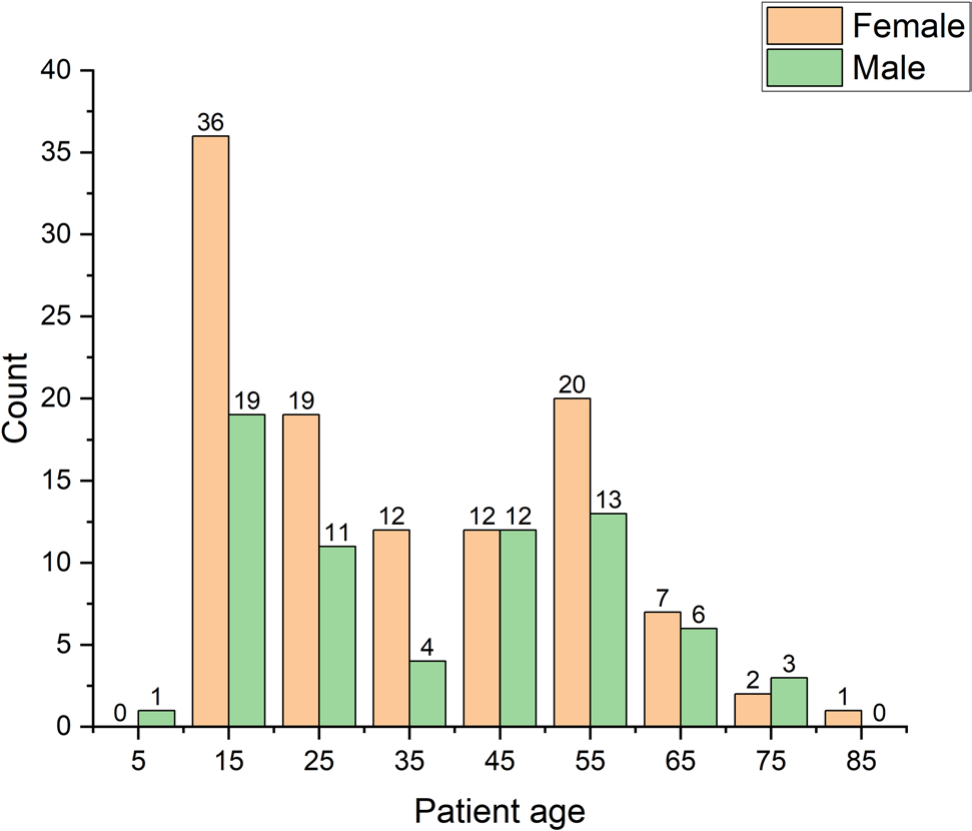
Age distribution of 178 scaphoid CT examinations in 174 patients

During the inclusion period, a total of 837 CT examinations of the wrist or hand had been recorded in the RIS (Fig. 1). Of these, 178 had been performed for suspect occult scaphoid fracture on 174 patients: 67 men and 107 women (Fig. 1), all referrals coming from the orthopedic clinic. One patient had had bilateral examinations, two had had examinations on the same wrist after repeated trauma more than six months apart, and one patient had had examinations of both wrists at separate time points. Median age at the time of examination was in 178 examinations 32.1 years (range; 8.5–81.1 years), for men 39.3 years (range; 8.5–78.9 years), for women 27.8 years (range; 10.6–81.1 years, Fig. 2). Totally 69 (39%) of the examinations had been done on men. The indications for the excluded 659 examinations are shown in Table 1.

**Table 1:** Indications for CT, other than suspect occult scaphoid fracture, in 659 patients

Indication	No. of studies
Non-scaphoid trauma: diagnosis and characterization	233
Postoperative control	83
Degenerative disease and pain	52
Distal radioulnar joint examination	27
Arthritis, tumor, developmental disorder	11
Research purposes	24
Scaphoid fracture position and healing control	160
Scaphoid fracture detected by radiography	2
Scaphoid fracture detected by MRI	2
Administrative reasons: not done, wrong code, double registration, etc	65
Total	659

### CT technique

All studies were performed on a Brilliance whole-body CT scanner (Philips, Eindhoven, The Netherlands) with 36 or 64 detector rows. Primary images were archived as 0.8-mm thick contiguous sections in the axial plane. In addition, 2 mm thick contiguous sections of the wrist in the three orthogonal planes were archived.

### Image analysis

The requests for CT and the CT reports were evaluated, and all available CT images were re-assessed in the picture archiving and communication system (PACS) to evaluate whether a scaphoid fracture was present or not. Fracture prevalence was analyzed according to age and sex. All further available clinical and imaging data for a period of minimum three years after the CT were then evaluated to determine whether the CT examination had missed a scaphoid fracture. The PACS archive contains all imaging studies for two university hospitals as well as for the surrounding eight hospitals within a radius of about 80 km, caring for a patient population of between 1.5 and 2 million people.

### Clinical history analysis

From the clinical records, the following data were collected: treatment type and duration, clinical follow-up, and complications. The clinical records were also perused for a period of a minimum of three years from the trauma to detect late presentation of occult scaphoid fractures.

### Statistics

Categorical variables are presented as frequencies and percentages. Comparisons between groups regarding differences in fracture prevalence between the sexes was done using the Pearson chi-squared test. *P*-values < 0.05 were considered statistically significant. All calculations were done using the R software package version 4.2.2 [27].

## Results

CT revealed 21 scaphoid fractures (12%) in 15 men (median age 27 years) and 6 women (median age 44 years). The time between initial normal radiography and CT was median 15 days (interquartile range (IQR) 20 days) and median treatment time with a scaphoid cast 40 days (IQR 42 days). While 15 of 69 examinations (22%) on men showed scaphoid fractures, only 6 of 109 examinations (6%) on women showed scaphoid fractures (*p* = 0.0024). Of all CT examinations showing a scaphoid fracture, 71% were done on men, while 66% of all examinations without fracture were done on women.

Median time from initial normal radiography to CT was in all patients 19 days (range; 0–125 days, IQR 20.5 days). In the 157 cases where CT showed no signs of scaphoid fracture, the median time between radiography and CT was 20 days (range; 0–119 days, IQR 16 days).

After a normal scaphoid CT, no late scaphoid fractures were revealed at imaging or clinical follow-up during the three year period of follow-up in the PACS and medical records. One case with scaphoid fracture had initially been reported as normal but was detected at the next-day morning conference and was treated without complications. The fracture was also detected in the current review prior to evaluating the radiology report. There was one equivocal CT examination which was followed up by MRI, revealing no fracture.

In 29 of 157 examinations showing no scaphoid fracture, several other fractures were detected at CT: 11 distal radius fractures, one lunate, eight triquetral, five trapezium, one trapezioid, one hamate, and two metacarpal fractures.

The forms of treatment of 157 patients without scaphoid fracture on CT, before and after CT, is shown in Table 2. At the review of the medical records, 135 patients (86%) had no remaining complaints. Nineteen patients with 20 examinations (13%) had various remaining symptoms (Table 3). In two cases, data were missing. Imaging follow-up in the 157 cases without scaphoid fracture on CT had been done in totally 28 cases (18%; Table 2).

**Table 2:** Clinical treatment and follow-up imaging in 157 patients with normal CT for suspected occult scaphoid fracture

		Scaphoid fracture	No scaphoid fracture
All patients		21	157
Men		15	54
Women		6	103
Treatment before CT			
	No treatment		24 (15%)
	Scaphoid cast		103 (66%)
	Other cast		21 (13%)
	Missing data		9 (6%)
Treatment after CT			
	No treatment		89 (57%)
	Scaphoid cast		24 (15%), median 14 days (4–59 days)
	Other cast		11 (7%), median 12 days (8–21 days)
	Removable splint		13 (8%)
	Surgery for distal radius fracture		2
	Missing data		18 (12%)
Follow-up imaging			28 (18%)
	Radiography		21 (13%), median 156 days (16–898)
	MRI*		12 (8%), median 85 days (8–539 days)
	No follow-up imaging		129

^*^ Five had both radiography and MRI

**Table 3:** Remaining symptoms in 19 patients with 20 CT examinations

Complaint	No. of patients	Comment
Carpal tunnel syndrome	6	7 CT examinations
Pain syndromes	6	
Osteoarthritis of 1st carpometacarpal joint	1	
ECU tendinosis	1	
Finger-hand-shoulder syndrome	1	
Scaphoid cyst	1	Later operated
Carpal collapse after scaphoid fracture some decades earlier	1	Operated
Reduced sensibility	1	
TFCC injury	1	Operated
Sum	19	

*ECU* extensor carpi ulnaris, *TFCC* triangular fibrocartilaginous complex

## Discussion

In the current study, there were no late complications from scaphoid fractures missed at CT. One scaphoid fracture was missed at initial reporting, but was detected the following day, and in the current study was detected on the blinded study review. Thus, it would appear that CT is a good enough method to evaluate suspect radiographically occult scaphoid fractures.

A missed occult scaphoid fracture has the potential for severe complications, such as AVN or pseudarthrosis, leading to SNAC. Every patient with pseudarthrosis will probably eventually develop SNAC, even after several decades. Therefore, all patients with remaining clinical signs of scaphoid fracture after radiography are usually treated with a cast until follow-up imaging has been done. This means that about 80% of patients with scaphoid trauma may be treated unnecessarily [28–30]. Recent guidelines [21] published after the patients in the current study were examined recommend early follow-up imaging with primarily MRI, stating CT as a reliable alternative in the absence of MRI, supported by the findings in the current study. A recent report analyzing the cost-effectiveness of various regimens suggests that immediate CT or MRI bypassing radiography are the most cost-effective ways to evaluate a suspected scaphoid fracture [31], something that has been implemented in many institutions.

MRI is usually advocated as the follow-up imaging of choice [14, 21] due to its high sensitivity. However, treatment of purely trabecular fractures at MRI may be unnecessary and potentially harmful, with possible complications arising from immobilization in cast. Differentiation between bone marrow edema and a true fracture with potential for displacement is difficult [26] with different studies having applied different definitions [16, 26]. A purely trabecular fracture without cortical fracture should not be regarded as a potentially unstable fracture, nor should an avulsion fracture of the scaphoid tubercle. Since the exact distinction between trabecular and cortical fractures at MRI has not been made, it would be unethical to perform a scientific study where scaphoid fractures at MRI are randomized to treatment with a cast for at least six weeks or to symptomatic treatment. The current study tries to address this predicament by using the approach that purely trabecular fractures at MRI would not show up at CT and were thus not treated more than symptomatically in the current study. None of these supposed cases showed progression into a displaced scaphoid fracture. However, since 124 of 157 patients wore a cast in the period preceding CT, and 48 patients were immobilized for a certain time after CT with a plaster cast or removable splint, it is not inconceivable that patients with non-displaced scaphoid fractures that had been occult on CT were included in this group. This treatment group also includes patients with other wrist or carpal bone fractures, patients with presumed ligamentous injuries, and patients receiving treatment for residual wrist pain. The combination of modern treatment, including wrist immobilization for pain until follow-up imaging and modern high-quality CT, thus seems adequate for diagnosis and treatment of suspected occult scaphoid fracture. During recent years, CBCT has come to be employed for primary diagnosis of scaphoid fracture in many institutions [17, 32], showing high sensitivity for scaphoid fracture at a lower radiation dose than imaging with a whole-body CT scanner [33], and having a higher spatial resolution. However, the number of suitable patients needs to be large enough to motivate the purchase of a specialized extremity CT.

During the study period, CT was the follow-up imaging of choice in case of suspect scaphoid fractures at the authors’ institution. At the end of the study period, increasingly more follow-up imaging was made with MRI. MRI has the potential ability to detect other reasons for post-traumatic radial sided wrist pain, such as other fractures or bone contusions, soft tissue contusions, and possibly ligamentous injuries [30]. In the current study, other fractures than a scaphoid fracture was detected in 29 patients without a scaphoid fracture, a prevalence of 18%. Other fractures than of the scaphoid have also been reported at scaphoid MRI [23]. A high prevalence of ligamentous injuries concomitant with scaphoid fracture has been reported at wrist arthroscopy [34]. It is also worth noting the sex difference in patient inclusion and fracture prevalence, with more women examined than men, but men having a significantly higher scaphoid fracture rate.

The strengths of the study are the large number of patients and long available follow-up time in the PACS archive and the medical records to detect possible complications from a missed scaphoid fracture on CT with later displacement. The limitations of the study are the retrospective nature, the lack of comparative imaging with MRI, not having dedicated scaphoid long-axis CT reformations, and not using dynamic multi-planar reformations (MPR) at the primary reading, which was not available in the clinical PACS at the time. A significant limitation which limits the conclusions to be drawn from the study is the fact that most patients received treatment with a plaster cast or a splint before CT, and many also for a time after CT as pain treatment, which makes it impossible to exclude that some non-displaced fractures may have healed during this treatment.

In conclusion, the current study revealed no missed occult scaphoid fractures at follow-up CT after normal radiography, suggesting that CT may be a reliable method for evaluating suspect scaphoid fracture as part of a diagnosis-treatment regimen including pain immobilization with a plaster cast. A randomized controlled study comparing the diagnostic value of CT and MRI treating only patients with cortical fractures at CT is warranted.

## Data Availability

The data sets generated and/or analyzed during the current study are not publicly available due to the General Data Protection Regulation (GDPR), but a limited and fully anonymized data set that supports the main analyses is available on reasonable request.
